# Supplementary material for: Antibacterial and Antiviral Effects of Ag, Cu and Zn Metals, Respective Nanoparticles and Filter Materials Thereof against Coronavirus SARS-CoV-2 and Influenza A Virus
Source: Pharmaceutics. 2022 Nov 22;14(12):2549. doi: 10.3390/pharmaceutics14122549 (PMC9785359; doi:10.3390/pharmaceutics14122549)
Supplement: Supplementary file 1 [file pharmaceutics-14-02549-s001.zip › pharmaceutics-2025094-supplementary.pdf]

Supplementary material for:

Antibacterial and antiviral effects of Ag, Cu, Zn metals, respective nanoparticles and filter materials thereof against coronavirus SARS-CoV-2 and influenza A virus

Anna-Liisa Kubo, Kai Rausalu, Natalja Savest, Eva Žusinaite, Grigory Vasiliev, Mihkel Viirsalu, Tiia Plamus, Andres Krumme, Andres Merits, Olesja Bondarenko

Table S1. Dynamic light scattering (DLS) for evaluation of nanoparticles in suspensions for producing filter materials.

Sample suspension	Time	NP	Solvent	% of polymer	Hydro-Dynamic size, nm	PDI
5%CuO_Ac:DMAc2:1	24 h	CuO	Ac:DMAc 2:1	-	254.5	0.3
17%CA_5%CuO_Ac:DMAc2:1	24 h	CuO	Ac:DMAc 2:1	17% CA	177.3	0.7
5%CuO_Ac:DMAc2:1	1 w	CuO	Ac:DMAc 2:1	-	221.8	0.3
17%CA_5%CuO_Ac:DMAc2:1	1 w	CuO	Ac:DMAc 2:1	17% CA	195.5	0.5
5%CuO-COOH_Ac:DMAc2:1	24 h	CuO-COOH	Ac:DMAc 2:1	-	3990.7	1.0
17%CA_5%CuO-COOH_Ac:DMAc2:1	24 h	CuO-COOH	Ac:DMAc 2:1	17% CA	320.4	0.4
5%CuO-COOH_Ac:DMAc2:1	1 w	CuO-COOH	Ac:DMAc 2:1	-	536.9	0.7
17%CA_5%CuO-COOH_Ac:DMAc2:1	1 w	CuO-COOH	Ac:DMAc 2:1	17% CA	258.8	0.3
5%CuO-NH_2_Ac:DMAc2:1	24 h	CuO-NH2	Ac:DMAc 2:1	-	715.4	0.8
17%CA_5%CuO-NH_2_Ac:DMAc2:1	24 h	CuO-NH_2	Ac:DMAc 2:1	17% CA	144.0	0.2
5%CuO-NH_2_Ac:DMAc2:1	1 w	CuO-NH_2	Ac:DMAc 2:1	-	394.3	0.6
17%CA_5%CuO-NH_2_Ac:DMAc2:1	1 w	CuO-NH_2	Ac:DMAc 2:1	17% CA	248.4	0.1
5%CuO_Ac:DMAc3:1	24 h	CuO	Ac:DMAc 3:1	-	288.2	0.5
17%CA_5%CuO_Ac:DMAc3:1	24 h	CuO	Ac:DMAc 3:1	17% CA	189.9	0.1
5%CuO_Ac:DMAc3:1	1 w	CuO	Ac:DMAc 3:1	-	288.9	0.4
17%CA_5%CuO_Ac:DMAc3:1	1 w	CuO	Ac:DMAc 3:1	17% CA	201.3	0.1
5%CuO-COOH_Ac:DMAc3:1	24 h	CuO-COOH	Ac:DMAc 3:1	-	10712.0	0.6
17%CA_5%CuO-COOH_Ac:DMAc3:1	24 h	CuO-COOH	Ac:DMAc 3:1	17% CA	232.7	0.4
5%CuO-COOH_Ac:DMAc3:1	1 w	CuO-COOH	Ac:DMAc 3:1	-	822.3	0.8
17%CA_5%CuO-COOH_Ac:DMAc3:1	1 w	CuO-COOH	Ac:DMAc 3:1	17% CA	432.1	0.3
5%CuO-NH_2_Ac:DMAc3:1	24 h	CuO-NH_2	Ac:DMAc 3:1	-	492.0	0.7
17%CA_5%CuO-NH_2_Ac:DMAc3:1	24 h	CuO-NH_2	Ac:DMAc 3:1	17% CA	199.6	0.0
5%CuO-NH_2_Ac:DMAc3:1	1 w	CuO-NH_2	Ac:DMAc 3:1	-	424.5	0.6
17%CA_5%CuO-NH_2_Ac:DMAc3:1	1 w	CuO-NH_2	Ac:DMAc 3:1	17% CA	200.6	0.3

NP- nanoparticle
w- week
DMAc- dimethyl acetamide
Ac -Acetone
PDI- polydispersity index
CA- cellulose acetate

Table S2. Operating parameters for electrospinning of the filter materials.

Samples	Additive concentration	Solvent system	Pumping rate	Needle diameter	Distance	Voltage
	(% w/w)		ml/h	mm	cm	kV
**CA**	-	Ac-DMAc (2:1)	0.6	0.4	15	10
**CA_7.5%CuSO$_4$**	7.5	Ac-DMAc (2:1)	0.9	0.6	15	10
**CA_10%CuO**	10	Ac-DMAc (2:1)	0.4	0.4	15	10
**CA_thymol**	10	Ac-DMAc (2:1)	0.4	0.4	15	10
**CA_thymol_7.5%CuSO$_4$**	10	Ac-DMAc (2:1)	0.4	0.4	15	10

Table S3. Physicochemical properties characterization of nanoparticles in suspensions.

Metal-based NPs or metal salts	Primary size, nm	Hydrodynamic diameter (Dh) in MQ water, nm (PDI)	Dh in RPMI cell culture medium, nm (PDI)	Z-potential in MQ water, mV	Z-potential in RPMI cell culture medium, mV	Metal content, %	Dissolution after 24h in RPMI cell culture medium, %
CuO	15.9 ± 5.2*	237 ± 31 (0.25)*	204 ± 13 (0.45)*	27.5 ± 1.8*	− 10.8 ± 1.4*	76.8 ± 5.7*	103 ± 0,5*
CuO−NH$_2$	6.9 ± 2.2*	733 ± 252 (0.24)*	936 ± 229 (0.67)*	25.8 ± 1.3*	− 8.9 ± 0.8*	46.2 ± 4.0*	99,3 ± 0,8
CuO−COOH	9.2 ± 2.5*	1124 ± 128 (0.35)*	303 ± 84 (0.70)*	− 12.0 ± 2.2*	− 10.2 ± 0.8*	33.6 ± 3.2*	98,9 ± 0,5
CuSO$_4$	na	na	na	na	na	37.1 ± 4.5*	102,9 ± 0,3
Ag-col	12.5 ± 4**	45.88 ± 0.21 (0.261)	61.2 ± 0.47 (0.24)	-56.6 ± 1.91	-9.76 ± 0.84	83.0 ± 9.8	5.24 ±0.41
nAg	85.7 ± 29.3	109.4 ± 1.3 (0.447)	156 ± 3.15 (0.403)	-27.7 ± 1.65	-10.49 ± 0.93	71.8 ± 12.0	1.1 ± 0.32
AgNO$_3$	na	na	na	na	Na	70.2 ± 7.95	96.7 ± 6.3

* Characterization of NPs has been done previously by *Kubo and al* (Kubo et al. 2020)

** Characterization of NPs has been done previously by *Blinova and al* (Blinova et al. 2013)

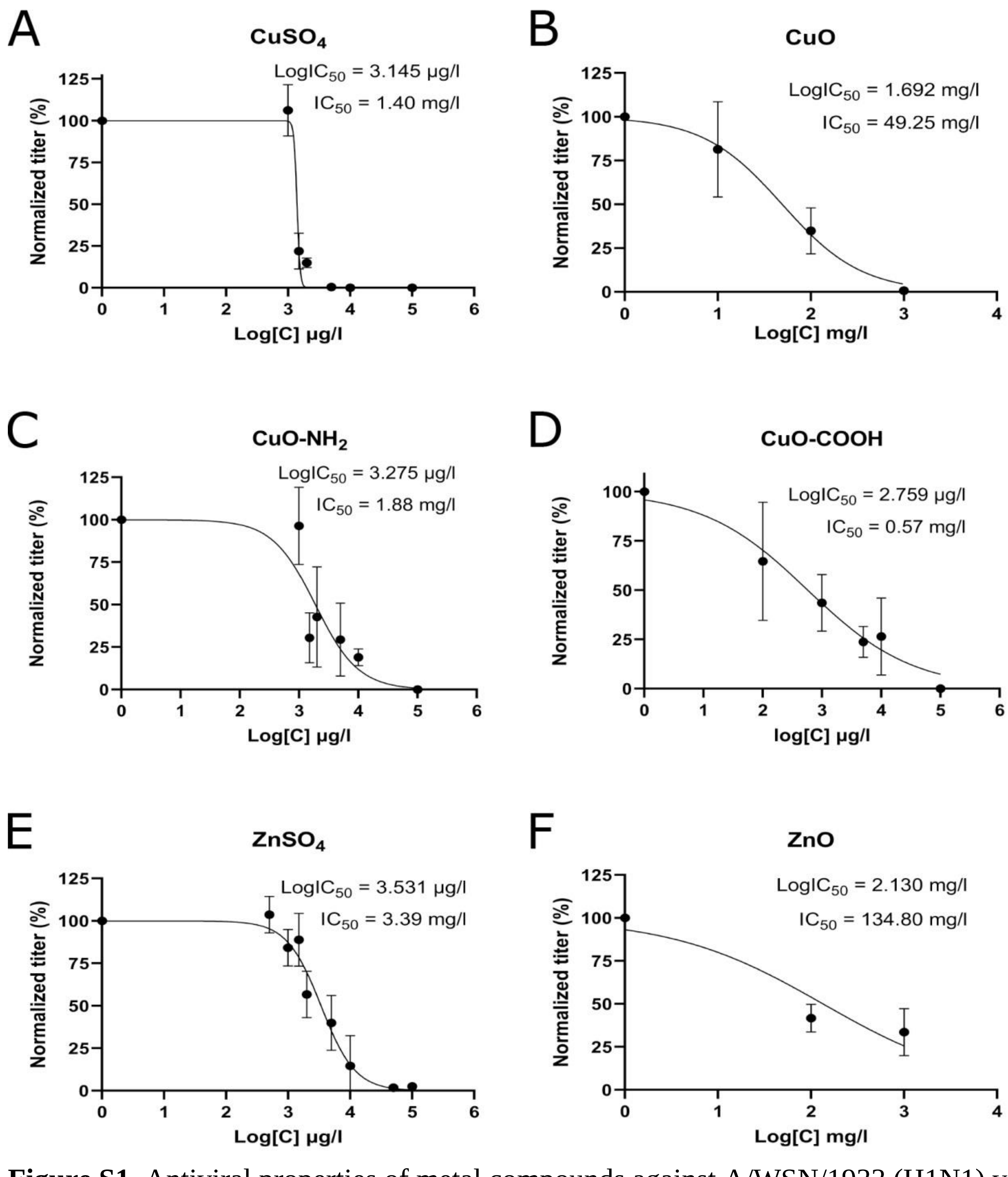

Figure S1. Antiviral properties of metal compounds against A/WSN/1933 (H1N1) virus in water suspensions. Note the differences in units (concentration of CuO and ZnO is shown in mg/l).

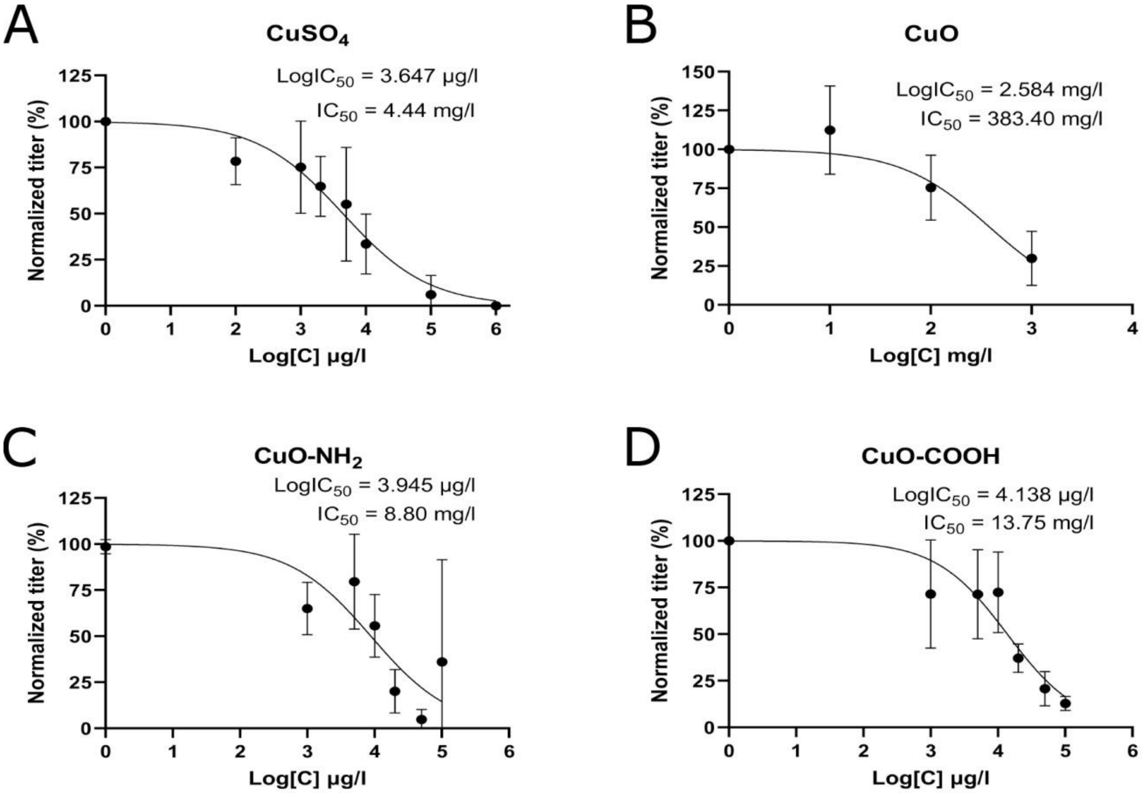

Figure S2. Antiviral properties of metal compounds against TGEV virus in water suspensions. Note the differences in units.

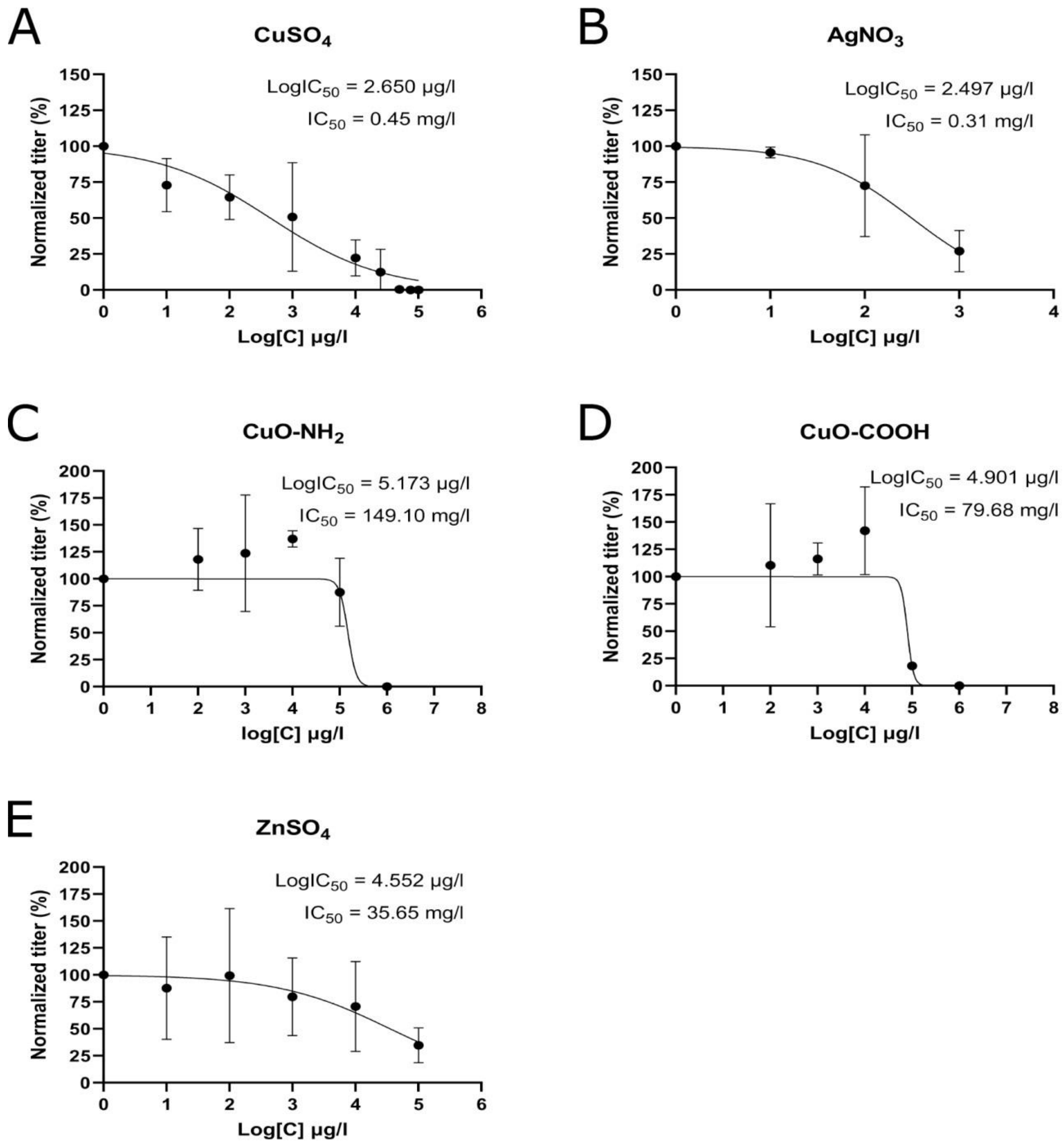

Figure S3. Antiviral properties of metal compounds against SARS-CoV-2 virus (Estonian strain 3049) in water suspensions.

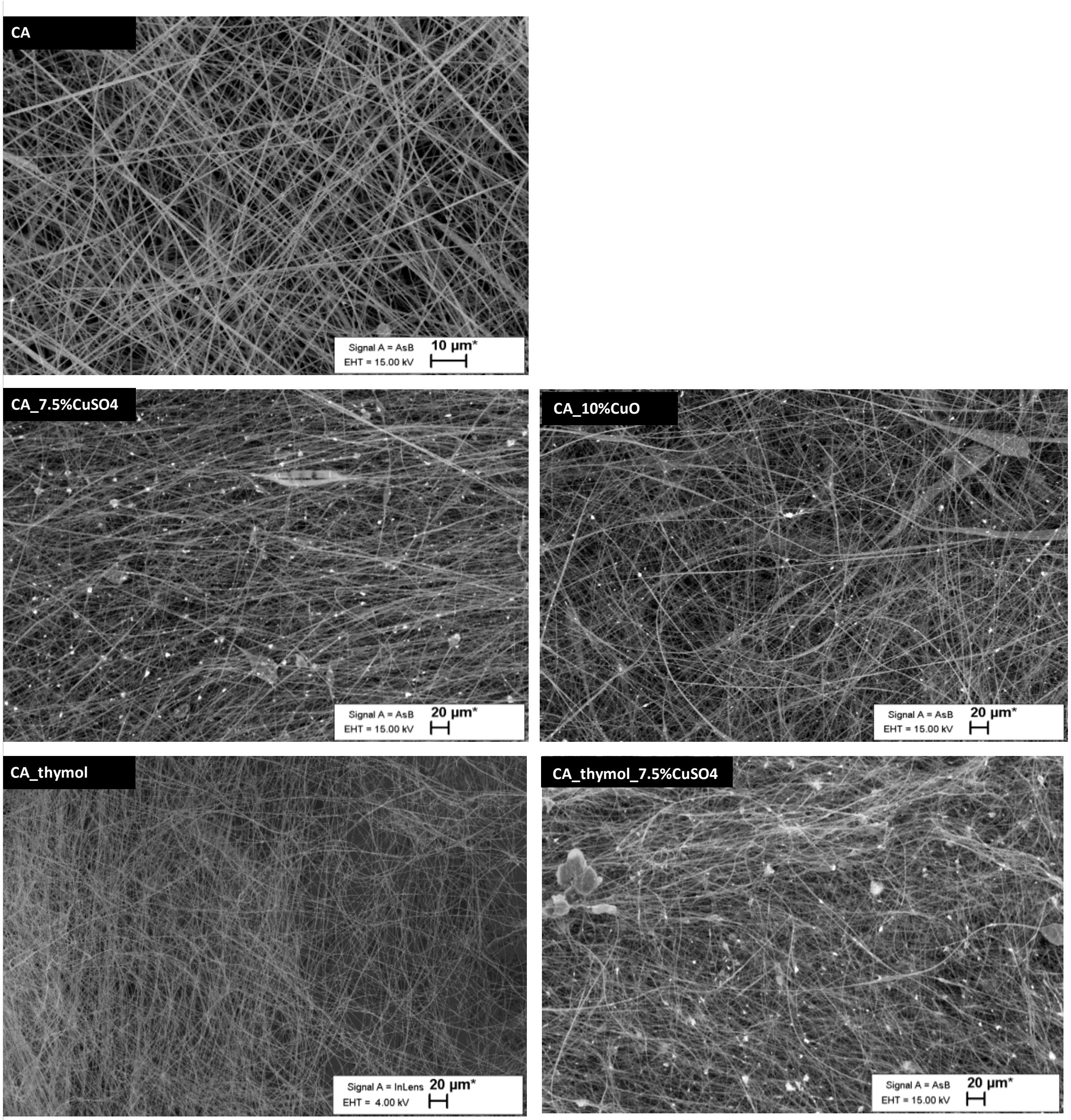

Figure S4. Scanning electron micrograph of CA fibers.

Literature for SM

Kubo AL, Vasiliev G, Vija H, et al (2020) Surface carboxylation or PEGylation decreases CuO nanoparticles' cytotoxicity to human cells in vitro without compromising their antibacterial properties. Arch Toxicol 94:1561–1573. https://doi.org/10.1007/s00204-020-02720-7

Blinova I, Niskanen J, Kajankari P, et al (2013) Toxicity of two types of silver nanoparticles to aquatic crustaceans Daphnia magna and Thamnocephalus platyurus. Environ Sci Pollut Res 20:3456–3463. https://doi.org/10.1007/s11356-012-1290-5
